# On the crucial importance of a small bird: The ecosystem services of the little auk (*Alle alle*) population in Northwest Greenland in a long-term perspective

**DOI:** 10.1007/s13280-018-1035-x

**Published:** 2018-03-07

**Authors:** Anders Mosbech, Kasper Lambert Johansen, Thomas A. Davidson, Martin Appelt, Bjarne Grønnow, Christine Cuyler, Peter Lyngs, Janne Flora

**Affiliations:** 1 Present Address: Department of Bioscience, Arctic Research Centre, Aarhus University, Frederiksborgvej 399, 4000 Roskilde, Denmark; 2 The National Museum of Denmark, Frederiksholms Kanal 12, 1220 Copenhagen, Denmark; 3 Greenland Institute of Natural Resources, Kivioq 2, P.O. Box 570, 3900 Nuuk, Greenland; 4 Christiansø Biological Fieldstation, Christiansø 97, 3760 Gudhjem, Denmark; 5 Department of Anthropology, University of Copenhagen, Øster Farimagsgade 5, 1353 Copenhagen, Denmark; 6 Department of Bioscience, Arctic Research Centre, Aarhus University, Vejlsøvej 25, 8600 Silkeborg, Denmark

**Keywords:** Ecosystem engineer, North Water polynya, Seabird ecosystem service, Seabird guano, Seabird cultural importance

## Abstract

The little auk is the most numerous seabird in the North Atlantic and its most important breeding area is the eastern shores of the North Water polynya. Here, a population of an estimated 33 million pairs breeds in huge colonies and significantly shapes the ecosystem. Archaeological remains in the colonies document that the little auk has been harvested over millennia. Anthropological research discloses how the little auk has a role both as social engineer and as a significant resource for the Inughuit today. The hunting can be practiced without costly equipment, and has no gender and age discrimination in contrast to the dominant hunt for marine mammals. Little auks are ecological engineers in the sense that they transport vast amounts of nutrients from sea to land, where the nutrients are deposited as guano. Here, the fertilized vegetation provides important foraging opportunities for hares, geese, fox, reindeer, and the introduced muskox. We estimate that the relative muskox density is ten times higher within 1 km of little auk fertilized vegetation hotspots.

## Introduction

While it is well described in the literature how marine mammals are the cornerstone of human life around the North Water (NOW) polynya (Born 1987), it is less recognized that a small seabird, the little auk (*Alle alle*), also has profound influence on human life and the ecosystem in the region (Gonzales-Bergonzoni et al. 2017). In this paper, we explore the benefits to people from the immense little auk population in the NOW region using the concept of ecosystem services. Ecosystem services have been defined as “the benefits provided by ecosystems that contribute to making human life both possible and worth living”,^1^ and the concept is often applied with a focus including ecosystem regulation, human provisioning, and cultural importance. We focus both on the ecological and social aspects of the harvest of little auks and on the indirect effect of the little auk as ecosystem engineer. Ecosystem engineers are organisms that directly or indirectly control the availability of resources for other organisms by causing physical state changes, and in that process, they modify, maintain, and create habitats (Jones et al. 1994). Ecosystem engineers have increasingly been recognized as relevant ecological drivers often leading to an increase in species diversity, but they are relatively rare in the Arctic (Romero et al. 2015). We will, in this paper, test the hypothesis that the little auk harvest is important for the local human population and that little auks significantly shape the terrestrial ecosystem and associated human possibilities due to their vast numbers. We take a broad view on the notion of social engineer, recognising that the little auk is but one of the many aspects that play into human social and cultural life, and of course, that the little auk is also “engineered” by “the social” (see also Ellen 1999).

**Fig. 1:**
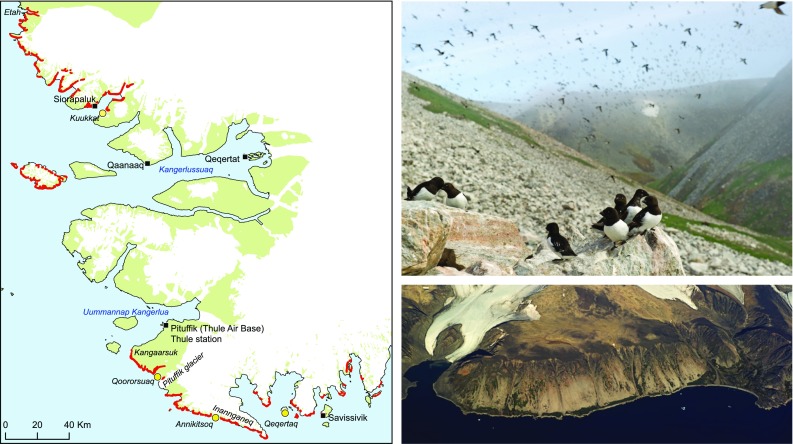
Illustration of little auk colonies and associated vegetation. To the left, the distribution of little auk breeding colonies (red areas) in the NOW area from Boertmann and Mosbech (1998). Inhabited settlements (black squares), little auk colony study sites (yellow dots) and place names referred to in the text are indicated on the map. Top right: Little auks gathering on a club stone in the Qoororsuaq colony (Photo: Peter Lyngs). Bottom right: Aerial view of a large little auk colony at Inannganeq (Cape York). The light grey area on the coastal slope is a high-density breeding area. Notice the barren areas inland of the colony and the green vegetated patches inside the breeding area and especially below the colony along the coast (Photo: Peter Lyngs)

The little auk is a small alcid (160 g) and the most numerous seabird in the North Atlantic (Barrett et al. 2006). The population has its most important breeding area in Avarnersuaq (Northwest Greenland, Thule district) on the eastern shores of NOW where an estimated 33 million pairs breed in huge colonies (Boertmann and Mosbech 1998; Egevang et al. 2003). The little auk colonies are generally located in screes on the coastal fringe but in some areas, they can extend up to 11 km inland (Fig. 1). The screes protect the nest, which is situated below stones, and each pair produces just a single chick during the 3-month breeding season from mid-May to mid-August. In the NOW region, the little auk is a summer visitor and only comes there to breed. It is a High Arctic specialist, which takes advantage of the particular ecological conditions of NOW, feeding on the rich marine zooplankton community (Petersen and Falk 2001; Karnovsky and Hunt 2002; Frandsen et al. 2014). During the breeding season, their chicks are mainly fed the large (up to 5 mm) lipid-rich copepod *Calanus hyperboreus* (Karnovsky and Hunt 2002; Frandsen et al. 2014), which is very abundant in the upper water layers of the eastern part of NOW in summer. Little auks feed their chick approx. 9 meals per day, each consisting of about 500 copepods brought in from the sea in a gular pouch (Frandsen et al. 2014).

In this paper, the direct and indirect importance to humans in the present and past is analysed based on ecological, anthropological, and archaeological fieldwork as well as ethnographic collections. In addition to the harvest related services, we have analysed how the little auk impacts the terrestrial ecosystem by bringing nutrients from the sea to land. We present a regional estimate of the transport of nitrogen from the marine environment to the terrestrial and freshwater environments. The impact of guano fertilization on vegetation near a colony was studied with vegetation plots along a transect, and the large-scale vegetation impact was studied using remote sensing satellite data. The importance for herbivores was studied by relating the spatial distribution of muskox to the areas fertilized by little auks.

## Materials and methods

In the analysis, we combine results from primary studies with published information, employing a variety of methods from different disciplines. The primary information on contemporary use and cultural importance of little auks comes from anthropological fieldwork in Savissivik and Qaanaaq in 2014–2016, looking at broader issues concerning hunting practices, climate change, and the social processes pertaining to the transformation of game to food. Using the ethnographic method of participant observation, which denotes observation through participation in social activities and the everyday, as well as unstructured interviews and conversations (cf. Spradley 1980; Okeley 2013), we were particularly concerned to explore the ways in which social dynamics change with the arrival of the little auk in spring. We obtained primary information on time budgets of little auks by GPS tracking. This allowed the estimation of terrestrial defecation, assuming that it is roughly proportional to time spent on land. Actively breeding little auks were caught by noose carpet close to their nesting site and fitted with miniature GPS loggers (Ecotone model Alle-60; Ecotone, Sopot, Poland; size 26 × 16 × 10 mm; weight 4.5 g; accuracy ± 50 m) with remote data download to a radio station placed in the colony. Loggers were attached to the central back feathers after no more than 15 min of handling and programmed to record a position every 15 min, starting 12 h after release or upon first dive registered by the on-board dive sensor. In total, 23 birds were tracked from three different colonies (Savissivik, Annikitsoq and Kuukkat), resulting in approx. 4400 positions, which we used to calculate time spent on land versus time spent at sea.

**Fig. 2:**
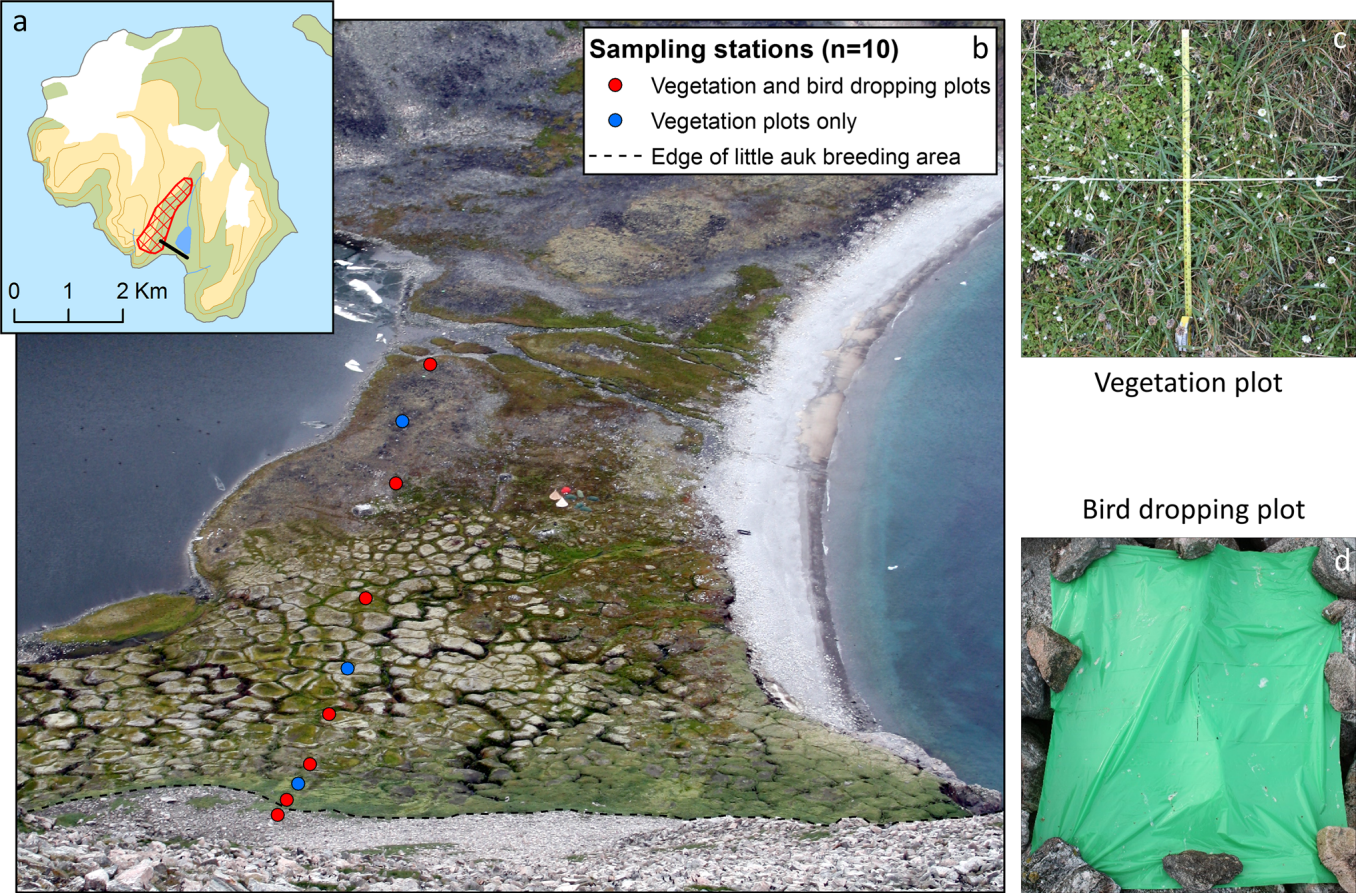
Vegetation study at Qeqertaq (Salve Ø) in 2014. A 350 m transect containing vegetation and bird-dropping plots ran perpendicular and down the slope from a high-density little auk breeding area to the river in the bottom of the valley. **a** Map of Qeqertaq with indication of the high-density little auk breeding area and the position of the transect. **b** Overview picture of the transect seen from the little auk breeding area. Stations (*n* = 10) with vegetation and bird-dropping plots are indicated (Photo: Kasper Lambert Johansen). **c** Example of 0.5 × 0.5 m vegetation plot (*n* = 62) (Photo: Anders Mosbech). **d** Example of bird-dropping plot (n = 7) (Photo: Kasper Lambert Johansen)

**Fig. 3:**
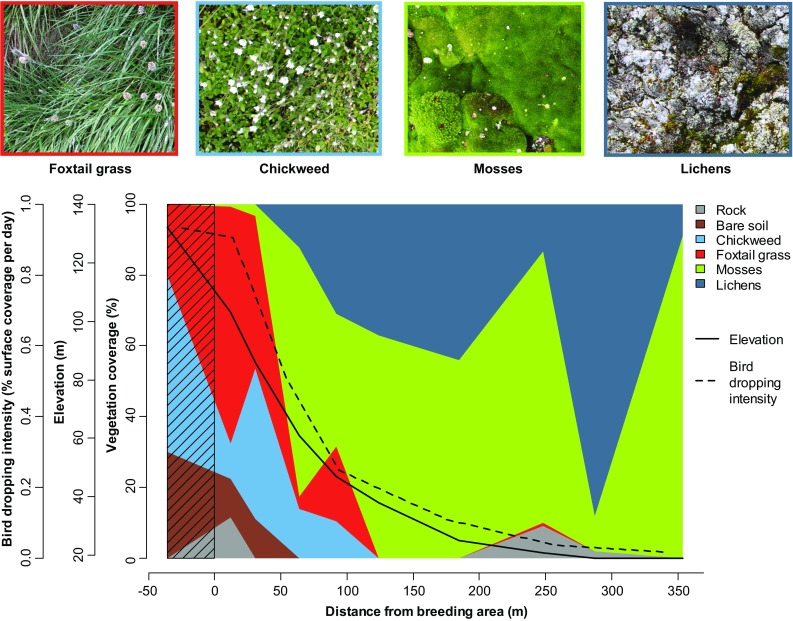
Change in plant communities with increasing distance from the little auk colony at Qeqertaq (Salve Ø). The x-axis designates horizontal distance from the little auk breeding area along the transect with 0 corresponding to the edge of the breeding area. The hatched area is inside the little auk breeding area and should in reality have 100% rock coverage (scree). Thus, the apparent vegetation cover here comes from a vegetation plot placed within an isolated green patch inside the breeding area. The three y-axes depict vegetation cover (colored polygons), elevation (solid line), and bird-dropping cover (dotted line). Photos of the four dominating vegetation classes are given above the plot (Photos: Anders Mosbech)

We obtained information on vegetation impact near a little auk colony at Qeqertaq (Salve Ø) where we described the vegetation in 62 0.5 × 0.5 m plots, located at ten stations on a transect extending approx. 350 m downslope from the main breeding area (Fig. 2). In each plot, the vegetation cover (%) was recorded in four gross categories: foxtail grass, chickweed, mosses, and lichens (Fig. 3). Subsequently, the vegetation cover in the plots of each station (5–10 plots per station) was averaged. At seven stations, we also placed plastic sheets to obtain a measure of bird-dropping intensity at different distances from the colony. Bird-dropping coverage (% area per 24 h) was estimated by extracting bird droppings on digital images of the sheets, using a supervised learning classification algorithm in ArcGIS 10.2 (ESRI, USA).

To examine the spatial extent of the vegetation impact caused by fertilization from little auk colonies, we analysed five Landsat-8 images from late July/early August 2015 (U.S. Geological Survey). The images had a spatial resolution of 30 × 30 m, and when combined, they covered the entire breeding range of the little auks around NOW. Initially, terrestrial areas (excl. inland ice, freshwater, and areas in deep shadow) were extracted using a supervised learning classification algorithm in ArcGIS 10.2 (ESRI, USA). For these terrestrial areas, Normalized Difference Vegetation Index (NDVI) values were calculated in ENVI 5.4 (Harris Geospatial Solutions, USA), using ATCOR2 (DLR, Germany) for atmospheric correction. To examine how little auk colonies influence vegetation productivity on a landscape level, we then binned the NDVI values (range − 1 to 1) in 0.1 intervals and calculated the average distance (± 1 SD) to nearest little auk colony of the cells in each bin, using information on the location of colonies from Boertmann and Mosbech (1998). To study the falloff in vegetation productivity with distance to colony, we also calculated the distance to nearest little auk colony of every 30 × 30 m terrestrial cell, binned the distances in 50 m intervals, and calculated the average NDVI value (± 1 SD) for each bin. Finally, to quantify the spatial extent of the little auk vegetation impact, we conducted a hotspot analysis (Getis and Ord 1992; Ord and Getis 1995) of the NDVI values, extracting all patches of the landscape significantly more fertile than the background level (95% conf. level; neighbourhood of 9 cells). Each identified hotspot was subsequently assessed as to whether it originated from influence of little auks or not, based on distance to nearest little auk colony registration, size and shape of hotspot, and landscape setting of hotspot. Due to partial cloud cover of some of the coasts in the northern part of the investigation area, the hotspot analysis was only completed for the southern part of the little auk breeding range in the NOW region (south of Uummannap Kangerlua (Wolstenholme Fjord)).

## Results

### The importance of the little auk in contemporary Inughuit society as food resource and social engineer

#### The food resource

**Fig. 4:**
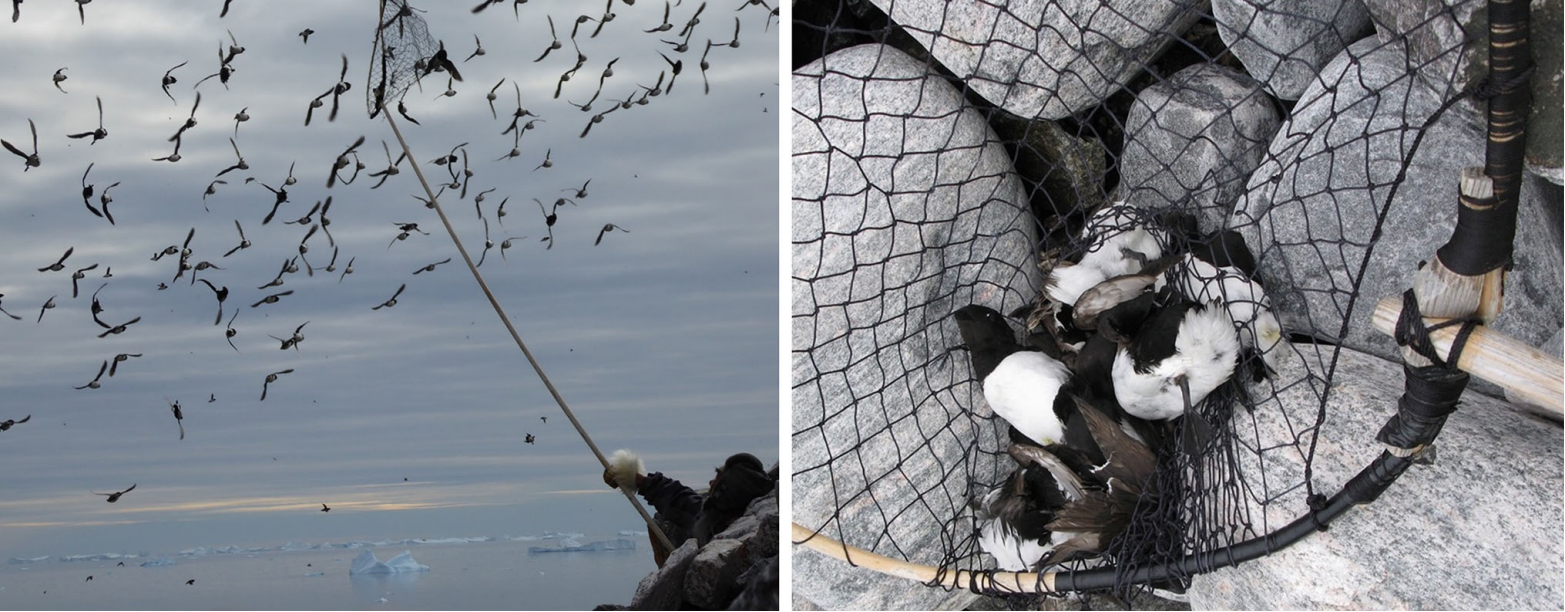
Inughuit hunting technique for harvesting little auks. To the left, a hunter catching little auks with ipoq from a hide in a little auk colony at Qeqertaq (Salve Ø) in the summer of 2014. To the right, a close up of the result of the hunt and the ipoq used for catching little auks (Photos: Anders Mosbech)

Based on the official harvest statistics from 1993 to 2013 (Piniarneq), the annual little auk harvest in the region has ranged between 7772 and 75 712 birds. There was a downward trend in the first 5 years (1993–1997, *r*^2^ = 0.49), while there was no discernible trend in the following 15 years which have had an average harvest of 16 912 birds and a large inter-annual variation (SD = 4955). Based on interviews with hunters, we estimated the annual harvest in the region in 2015 and 2016 to be roughly 40 000 adult little auks and 1000 eggs. The large difference between Piniarneq and our estimate is most likely because the interviewed hunters had a larger harvest than the average hunter, and it is also possible that there has been some underreporting to Piniarneq due to the diverse harvest pattern (see below). Most likely, the true harvest is between these two figures. Compared to an estimated population of 33 million pairs, this harvest is negligible, even though the harvesting of adults during breeding has extra population costs (the chicks). Due to the vast and dispersed colonies, and the hunting practices employed, we estimate that the provisioning of little auks have only a minor and local impact on the little auk population, although there is no quota or other restrictions on the harvest and no population monitoring to produce a population trend. The little auk is mainly harvested using an *ipoq* (a net tied of string, mounted onto a circular frame, and attached to a long rod) when they fly low over the colony (Fig. 4). The little auks circle over the colonies both on their way out to sea and when they return. Often, they make a low pass over the colony and can be snatched with the *ipoq*. Regardless of nest location, birds will pass low over much of a colony, and therefore, the harvest pressure is distributed over several hundred meters, even if the hunting hides are concentrated in certain areas.

Based on interviews, it is estimated that the local consumption of little auk meat ranges between 1 and 10% of the individual meat consumption, though this varies greatly from household to household, region to region, and according to the time of year. Little auk meat is among the least contaminated of the local food sources. Due to the low trophic position as a plankton feeder, little auk meat contains less mercury (Hg) than the main meat sources, i.e., ringed seal (*Pusa hispida*) and narwhal (*Monodon monoceros)* (Dietz et al. 2018).

#### The little auk in social networks: A social engineer?

Though meat from little auks constitutes only a small percentage of the total amount of meat consumed for most people in the region, the harvest has significant cultural bearings, especially in Savissivik, which is located immediately at the foot of a talus slope with a large colony of little auks. Here, little auks are in easy reach for anyone to catch, whenever needed. The arrival of the little auk to Savissivik in late April marks the beginning of a new season in environmental and social terms alike.

Their arrival instigates a new social rhythm, giving rise to a change in activities and an involvement of the whole community in hunting activities. Anyone who is able bodied and strong enough to manoeuvre the *ipoq* can catch little auks. The youngest children tend to gather eggs in the nests located under the small clusters of rocks at the foot of the slope, where there is little risk of causing an avalanche. For the elderly, who might once have lived from hunting and thus enjoyed much mobility, but today have become dependent upon their relatives, this season allows them a greater sense of self-sufficiency and personal agency. In this way, the little auk emerges not just as a food resource, but as a kind of social engineer in its own right.

While the design of the *ipoq* is ancient, the material used to make it has changed entirely. Instead of tying nets out of string made from meticulously treated sinews or baleen, people use imported nylon string that can be bought in the shop. The shaft is now a long and light bamboo rod, also imported and purchased in the village shop, and the circular frame onto which both the net and the rod are fastened is now made of bamboo as well. Some people even use plastic hula-hoops, but essentially the technology and method of catch remains the same.

Many have their own favourite spots from where they enjoy catching little auks. Such places may have a particularly good view or they may be comfortable, or they may be endowed with particular personal memories and important, because they have been in use for generations. Such favourite places are recognizable, since their users have fashioned little rock walls, behind which they can remain concealed from the little auks as they swerve low in circular motion in large flocks over the slope. When the birds are close enough, the hunter swings his/her *ipoq* into the air in a sideways motion, while twisting the *ipoq* 45 degrees downwards, thus capturing any birds that got into the net. In what seems like one swift and flowing movement, the hunter pulls in the *ipoq,* removes the bird from the net, and twists its wings together on its back, while firmly pushing a thumb into the side of the bird, directly on the heart, instantly shutting off the blood circulation. Within a brief moment, the bird is dead, and placed in a bag that is kept out of direct sunlight.

Almost everyone knows which favourite places are used by whom, but no one can claim a place as their personal property. They can be used by all as long as they are not in use. Some families prefer to catch little auks in other colonies than the one in Savissivik, such as the nearby island Qeqertaq (Salve Ø) located between Savissivik and Inannganeq (Cape York).

The most remarkable dish prepared with the little auk is the *kiviaq.* The *kiviaq* is made in spring, using a freshly caught seal and freshly caught birds. The birds, including feathers and all, are stuffed inside the seal pelt, on which a thick layer of blubber remains. Before the seal pelt is stitched together, enclosing all the birds, the air is stamped out entirely to ensure that the birds do not go bad while undergoing fermentation. There are regional differences to making *kiviaq*, and the flavour of it is also greatly impacted by the landscape, where the *kiviaq* is buried, thus interweaving both the physical attributes of the landscape, the weather and temperature conditions, *terroir*, with the psychological and historical attachment people may have to a place. All families have their favourite places to make *kiviaq*, and many use the same rock cache that their parents and grandparents may have used. Today, people eat the *kiviaq* mainly at times of celebration, and as people prepare them, they plan ahead and make enough *kiviaq* to eat at upcoming special events.

In the spring and summer, when little auks are caught fresh, people frequently enjoy eating them prepared “in the coat” *amilik*. The birds, again feathers and all, are covered with water and ample salt in a large saucepan, and weighted down with rocks to ensure they cook evenly and do not float atop the water. This method of cooking lends itself especially to the outside, in summer camps at the foot of a bird colony. Another way of preparing the bird is with *suaassat* (rice soup), using only the breast meat (with bone) and the heart, which have been separated from the rest of the bird. Hanging the breast meat (with bone) side by side on a long string on a drying rack, it is also suitable for making *nikku* (dried meat).

The little auk harvest circulates throughout the district along formal and informal human networks and as with all other meats, the price of a little auk has been universally agreed upon by the women’s association and hunters’ association, so as to avoid barter and situations in which individuals over charge or undercut (in 2015 1.3 USD per fresh bird, and 1.7 USD per fermented bird, although this depends upon season). For parts of the year, the circulation of the little auk harvest thus plays a role in the shaping of social networks, binding people and even the entire Avanersuaq district together, and mitigates the effects of a demographic and economic separation of the inhabited parts of the region. Owing to the depopulation and abandonment of Uummannap Kangerlua (Wolstenholme Fjord) in the middle of the district, there is a widespread understanding of the Avanersuaq district as broken in two, and that Savissivik is separated from the region’s centre, Qaanaaq, and the nearby villages Siorapaluk and Qeqertat in Kangerlussuaq (Inglefield Bredning) (see also Flora et al. 2018). Over the recent decades, many people from Savissivik have settled in Qaanaaq either permanently or for parts of the year. The tendency of outward migration from Savissivik is abruptly counterbalanced in spring after the arrival of the little auks to Savissivik. It is not long before people in Qaanaaq, who originally came from Savissivik, begin to orient themselves southwards, talking longingly about the little auks that have arrived, some even making plans to return to Savissivik for spring and summer. Many Qaanaaq residents make use of their contacts in Savissivik to purchase little auks from there, and soon shipments of little auks begin to arrive. Thus, in some ways, Savissivik temporarily becomes the center of the region by way of the little auk’s presence.

### The little auk as ecosystem engineer: Fuelling the terrestrial ecosystem

#### Transport of nutrients from the sea

The little auk colonies are located in screes in the coastal fringe; however, in contrast to the other seabird colonies in the region, the little auk colonies in some areas extend up to 11 km inland (Fig. 1) and their guano or droppings can thus fertilize a substantial area. GPS tracking of little auks illustrate that the marine feeding areas extend up to 100 km from the colonies, while the main feeding areas during chick rearing seem to be about 10 km from the colony. From arrival in early May to fledging and departure in mid-August, they transport nutrients to the colony and its surroundings through feeding of the chicks and defecation in the colony and while flying over land. The GPS tracking reveals that during chick rearing, little auks spend about 25% of their time in the colonies, circling, attending the chick, and resting.

The food intake, and thus the nutrients passing through the little auks, can be estimated based on the metabolic rate. The little auk has the highest metabolic rate of any alcid relative to their size (Gabrielsen et al. 1991). Using double labelled water, the total Daily Energy Expenditure (DEE) has been estimated to 598 kJ/d in a year with poor feeding conditions, and 757 kJ/d in a year with good feeding conditions in a Svalbard colony (Welcker et al. 2009a). Using the average DEE, an assimilation efficiency of 80% (Taylor and Konarzewsky 1992), and an energy density of Calanus of 26 kJ/g dry weight (dw) (Weslawski et al. 1994), it is estimated that an adult little auk consumes 32 g/d (dw), roughly equivalent to its own weight (160 g in wet weight (ww)). While some of the carbon is respired for energy consumption, the total nitrogen has a balanced food intake and guano output for an adult bird. The nitrogen (N) content in the copepods is 8% (dw) (Hildebrandt et al. 2014). As an adult bird spends 25% of its time in the terrestrial environment during a 3-month breeding season, we can assume that 25% of the droppings fall on land. This results in a total annual N load to the terrestrial environment of 115 g per little auk breeding pair.

$$ {\text{Daily intake }}\left( {\text{g dw}} \right) \, *{\text{breeding season }}\left( {\text{d}} \right)*{\text{ terrestrial dropping fraction (\%)\,}}*{\text{N content in copepods }}\left( \% \right). $$

To this amount comes the N contribution from the chick which is fed about 650 g ww (25 g ww/d in 26 days, Frandsen et al. 2014) before it fledges at a weight of about 110 g (ww) and thus leaves behind about 7.6 g N.

$$ \left( {{\text{total intake}} + {\text{egg weight}} - {\text{fledging weight}}} \right){\mkern 1mu} *{\text{ N content }}{\text {in copepods}}\left( \%  \right).  $$

We assume a 75% breeding success. However, for the population of 33 million pairs (Egevang et al. 2003), we estimate the input based on only a 10% reduction (30 million successful pairs), to account for the input from the failed breeders and non-breeders which also frequent the colony. This gives a total estimate of 3645 tons of N per year transported from the sea to the terrestrial environment.

#### Vegetation impact near a little auk colony

The transect at Qeqertaq (Salve Ø) shows how the vegetation develops as a function of distance from a high-density little auk breeding area (Fig. 3). Variance is large, because the vegetation is patchy, but there is a clear progression of vegetation types. Immediately below the breeding area, there is a zone dominated by foxtail grass and chickweed. These are also the species that characterize isolated green patches inside the breeding area. At distances greater than 50 m away from the breeding area, the foxtail grass and chickweed gives way to mosses, which remain dominant for the rest of the transect. However, lichens, which mainly grow on top of mosses, also become more and more significant, so on an overall level, there is a foxtail grass/chickweed zone and a moss/lichen zone. This progression of vegetation types is probably mainly explained by different drainage conditions: The foxtail grass/chick weed zone is found up high on the steep and well-drained slope, whereas the mosses are found lower down on the flat and relatively wet areas (see elevation curve in Fig. 3). Similar vegetation patterns were also observed at Annikitsoq and Savissivik, and seem to be characteristic of little auk colonies in Thule.

The bird-dropping intensity along the transect increases dramatically close to the breeding area (Fig. 3). In, and immediately below, the breeding area, approx. 1% of the surface area, is covered by bird droppings every 24 h. This means that the foxtail grass/chick weed zone receives a much higher direct input of nutrients than the peat/lichen zone. However, we assume that the nutrients also seep downslope from the breeding area with water from rain and snowmelt, so nutrients are spread much further than suggested here by the bird-dropping intensity.

The vegetation description was kept in rather general classes. However, the low number of classes does reflect that while productivity is high, plant species diversity is fairly low in the little auk colonies. Both foxtail grass and *Aplodon wormskioldii*, which dominates amongst the mosses, are well known for favouring highly enriched habitats^2^ (Bennike et al. 2008).

#### Large-scale vegetation impact

**Fig. 5:**
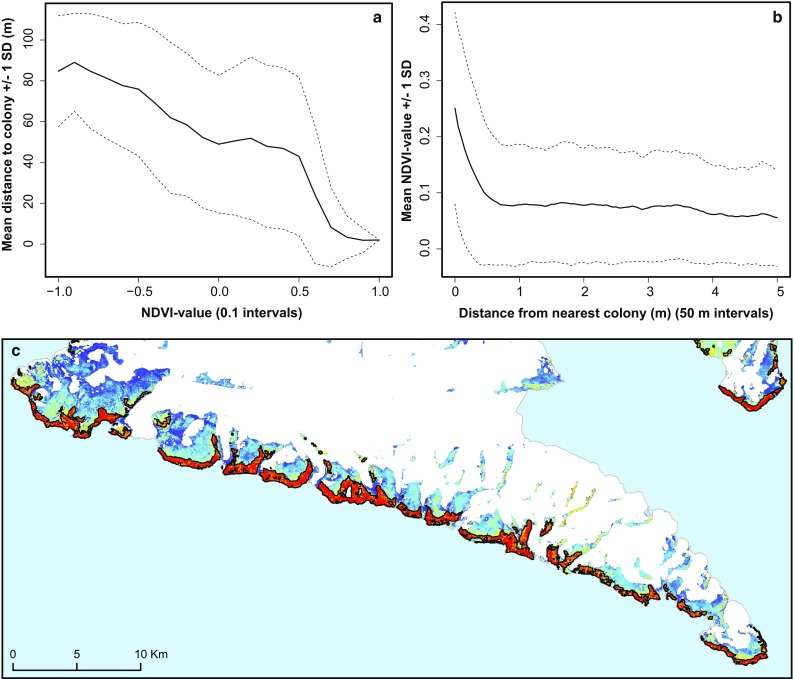
Relationship between landscape “greenness” and little auk colonies based on analysis of 30 × 30 m NDVI data from Landsat-8 images. **a** The average distance (km, ± 1 SD) to nearest little auk colony of NDVI values of different of different sizes (0.1 intervals). It is evident from the graph that NDVI values > 0.6 are almost exclusively found close to little auk colonies. **b** Average NDVI value (± 1 SD) at different distances from little auk colonies (50 m intervals). It is only really within 600 m of little auk colonies that the average NDVI value is above the landscape average. **c** NDVI map of the Inannganeq (Cape York) peninsular on a color scale from blue (barren) to red (fertile). White areas correspond to inland ice. Little auk induced vegetation hotspots, resulting from the hotspot analysis, are indicated with a black outline. As can be seen from the map, the vegetation of almost the entire coast is transformed by little auk presence. In fact, approx. 25% of the terrestrial landmass of Inannganeq has elevated vegetation productivity due to little auk colonies

We used satellite data to examine the spatial extent of the vegetation impact of little auks. The results showed that nearly all the most fertile/green areas in Thule (NDVI values > 0.6; NDVI is a greenness index) are found in the vicinity of little auk colonies (Fig. 5a). However, not all areas close to little auk colonies are green. The birds use very specific flight paths between their colony and at-sea foraging areas, and it is only areas under that flight path, and areas that receive drainage water from the breeding area or the flight path, that are green (Gonzalez-Bergonzoni et al. 2017). It is only within 600 m of the colonies that the average NDVI is clearly above the landscape average (Fig. 5b). Thus, the landscape impact of little auks does not become extensive, because they affect a big area around individual colonies, but more so because there are so many colonies with local impacts spread along the coasts of NOW. To estimate the total area of the vegetation impact of little auks, we conducted a hotspot analysis of the NDVI data (Fig. 5c; see Methods). Through this analysis, we identified patches (hotspots) of the landscape significantly more fertile/green than the background level. In the southern part of the NOW region, south of Uummannap Kangerlua, we found more than 85 km^2^ of terrestrial vegetation hotspots, which could be attributed to the presence of little auk colonies (hotspots overlapping or ≤ 1 km from a little auk colony). For the northern part of the NOW region, we could not complete the same analysis due to clouds on the satellite images. However, in total for the NOW region, we estimate based on extrapolation that little auks are responsible for creating > 200 km^2^ worth of “terrestrial oasis” in the otherwise barren High Arctic landscape.

#### The trophic cascade: The Kangaarsuk muskoxen as example

**Fig. 6:**
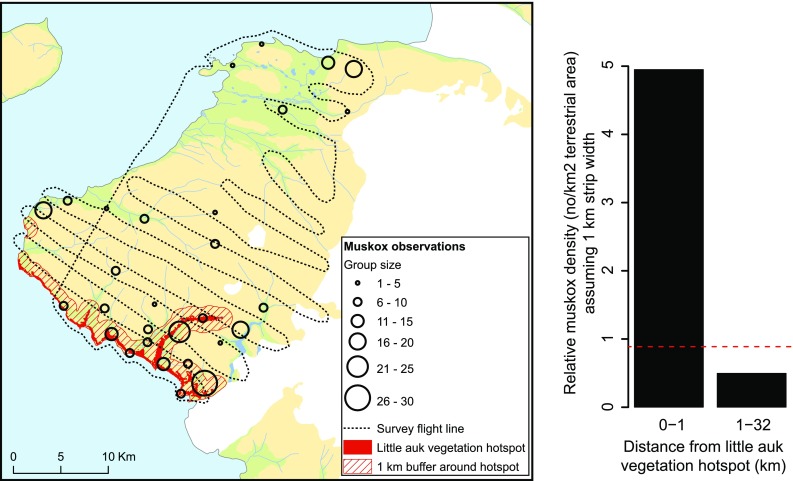
Distribution of muskoxen relative to vegetation hotspots caused by little auk colonies in the Kangaarsuk (Cape Atholl) area. In the map to the left, muskox sightings from an aerial survey conducted in early September 2015 (open circles; from Cuyler et al. 2016) are combined with the distribution of little auk induced vegetation hotspots (red polygons; see Methods) and a zone of 1 km around these hotspots (red hatched area). The bar plot to the right compares the relative muskox density (adults and calves) inside and outside the 1 km zone around little auk vegetation hotspots (dotted line is the average relative density for the whole area). As can be seen, the relative density of muskox is almost 10 times higher in proximity to little auk vegetation hotspots

**Fig. 7:**
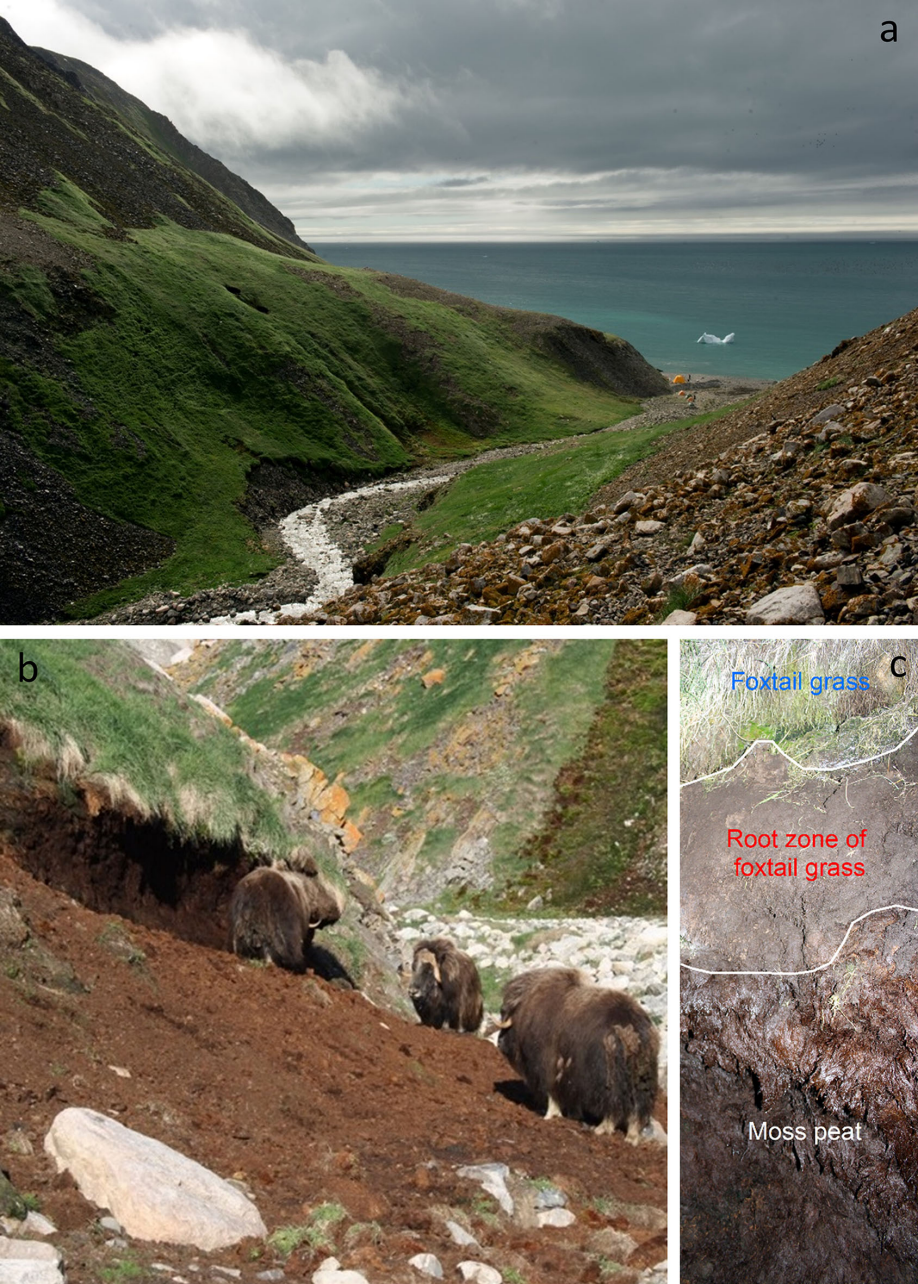
Muskoxen altering the landscape. Heavy muskox presence in the little auk colony in Qoororsuaq has transformed the vegetation from being dominated by mosses to being dominated by grasses, the valley almost taking on the appearance of a pasture (**a**). This transformation of the landscape is evident in areas where the muskox rub against the soil (**b**). In the sections hereby exposed, it can be observed that the current vegetation, completely dominated by foxtail grass, grows on top of a thick, homogenous deposit of mosses (**c**) (Photos: Kasper Lambert Johansen)

The little auk is important in the terrestrial ecosystem as prey for glaucous gulls *(Larus hyperboreus),* gyrfalcons *(Falco rusticolus),* and Arctic foxes (*Alopex lagopus*). However, the landscape engineered by the little auks is also an important habitat for several grazers: Arctic hare (*Lepus arcticus*), geese, reindeer (*Rangifer tarandus*), and muskoxen (*Ovibus moschatus*). Here, we take the muskox population of Kangaarsuk (Cape Atholl) as an example. Muskoxen had been absent from Kangaarsuk for about a century, when seven 1-year old muskoxen (5 females and 2 males) were re-introduced in 1986 (Vibe 1986). They were released just north of the Pituffik Glacier, in a valley with little auk colonies, and the population thrived on the lush vegetation associated with the birds (Burnham 1996, 1997; Cuyler and Mølgaard 2002). In 2015, the muskox population was surveyed from airplane (Cuyler et al. 2016), and we used data from this survey to examine the relationship between muskox distribution patterns and vegetation hotspots caused by little auks. In total, 273 muskoxen were observed during the aerial survey (Fig. 6). A spatial analysis, comparing a zone of 1 km around little auk induced vegetation hotspots with the rest of the surveyed area, reveals that in proximity to little auk vegetation hotspots, the relative density of adult muskoxen is almost ten times higher (3.9 vs. 0.4 ind/km^2^ assuming 2 × 500 m coverage around the flight line; Chi squared test: *χ*^2^ = 414.92, df = 1, *p* < 0.001), the relative density of muskox calves is almost 15 times higher (1.03 vs. 0.07 ind/km^2^ assuming 2 × 500 m coverage around the flight line; Chi squared test: *χ*^2^ = 147.36, df = 1, *p* < 0.001), and the average muskox group size is almost twice as high (13.5 vs. 7.4 ind/group; GLM: dev.exp = 0.18, df = 27, *p* = 0.024). Thus, there is no doubt that the highly productive vegetation associated with the little auk colonies is important for the Kangaarsuk muskox population. Since their introduction in 1986, the muskox have remained north of the Pituffik Glacier and concentrated in vicinity of the little auk colonies. The constant trampling and substrate disturbance of the vegetation for more than 30 years has altered the plant community from mosses towards grass meadows. The characteristic vegetation zones associated with little auk colonies described for Qeqertaq (Salve Ø), where there are no muskoxen (Fig. 3), are not evident where there is a heavy muskox presence. At the Qoororsuaq colony, where muskox density is high, foxtail grass is completely dominant, and it has clearly expanded into areas that were characterized by mosses before the muskoxen were introduced (Fig. 7). Apparently, the mosses are unable to tolerate the traffic by the muskoxen and become outcompeted by the foxtail grass, which tolerates trampling and is stimulated by muskox grazing. Thus, the muskoxen promote their own possibilities for grazing at the little auk colonies. They are also engineers, changing the plant community to their own advantage.

### The significance of the little auk in the past

#### Harvest of little auks in the past

The human history of little auk harvest in the NOW area is a tale of a back-up resource that for some periods and parts of society, in different ways, becomes a primary resource. We know from peat and lake cores that the little auk colonies are at least 4400 years old (Davidson et al. 2018), which coincides approximately with the first human settlement in the area. Archaeological remains and ethnographic sources document that the little auk has been important for human societies through centuries (Gilberg 1994; Johansen 2013 and references therein; LeMoine and Darwent 2016). Little auks added resilience to human societies in times when marine mammals were difficult to access due to sea ice conditions. Little auks had particular importance in the late 18th century and the first half of the 19th century when seafaring technologies (kayak and umiaq) were lost by the Inughuit. This made marine mammal hunting difficult in the open water season, and it promoted a heavier reliance on the little auk, which is available at this time of the year (Johansen 2013; LeMoine and Darwent 2016 and references therein).

The archaeological evidence of human presence in the North Water area during in the later parts of the 17th century and most of the 18th century is scant, and absent on the Canadian side. One exception though is the Etah (also spelled Iita) site in Foulke Fiord (southernmost Inglefield Land) that is situated immediately below a massive colony of little auks. Recent excavations seem to suggest a continuous use of the site from Late Dorset (8th–13th century AD) until final abandonment in the late 20th century (LeMoine and Darwent 2016). The Etah site is also the only site in the area where bird bones account for 75% of all the fauna material excavated, thus significantly contrasting the average 1–3% bird bones recorded on other Late Dorset and Inuit sites (Johansen 2013). A second example in which the little auks constitute a primary resource is from the early part of the 20th century. In his 1921-geographical portray of the Thule District, Knud Rasmussen described the land north of the Pituffik glacier as possessing “mild valleys where the Polar Eskimos left the elders of the tribe from spring until autumn. It was a kind of high mountain resort, where everybody could easily fend for themselves, by hiding amongst the rocks, catching the birds (little auks) as they flew to and from the nests” (Rasmussen 1921, p. 531, author’s translation). The archaeological remains from one of these “mild valleys”, Qoororsuaq, are described in Box 1.

**Table:** 

**Box 1** Qoororsuaq	
Since 2010, a large little auk breeding colony in the valley of Qoororsuaq has served as study site for a range of investigations into little auk breeding ecology (Frandsen et al. 2014; Mosbech et al. 2017). Located on the coast approx. 3.5 km north of the Pituffik Glacier, Qoororsuaq is remote from present day settlements. However, it soon became clear that Qoororsuaq was rich in archaeological remains, testifying to a period of intense human use in the past. In 2012, approx. 180 archaeological features were located by surveys on foot and recorded by descriptions, metric properties and photos. While these surveys do not cover the total valley system, the recorded features are sufficient to demonstrate that Qoororsuaq is a unique site. It allows a glimpse of the life in a bird hunting camp, which was probably used until the end of the Thule Station Period, i.e. about seventy years ago. The spatial distribution of features in Qoororsuaq is strikingly different from most Thule Culture/early historic sites in that most features are located quite far from the coast (Fig. 1). Progressing inwards from the coast, we first encounter a cluster of caches on a small plateau. Disassociated from other archaeological remains in the valley, these probably represent a dedicated storage area intended for easy pick-up when travelling by dog sledge on the sea ice. Further up the valley, numerous hunting blinds are recorded scattered within the actual breeding area of the little auks. The recorded hunting blinds represent merely fraction of the total number, and a systematic survey would probably reveal the slopes of the valley to be littered with these features. It is only once we get approx. 900 m up the valley that we come across a regular settlement. This site, which exclusively contains dwellings from the warm season, is strategically placed on a flat plateau opposite to where the valley branches off to the south and thus allows sunlight to penetrate into the valley. Another distinct settlement area, which in addition to dwellings from the warm season also contains two winter houses, is located 500 m further up the valley system. Just as the spatial distribution of features sets Qoororsuaq apart, so do the feature types (Fig. 1). The hunting blinds, which consist of small pits and/or associated stonewalls, erected within the boulder scree where the little auks nest, are characteristic of the site. Today, hunters from Savissivik and Siorapaluk use similar structures to hide behind when netting little auks with *ipoq*, fragments of which have also been observed in Qoororsuaq. Another hallmark of the site are the numerous caches, which with average chamber dimension of 68 × 49 cm (*n* = 46) are substantially smaller than caches normally encountered at sites focussed on hunting of marine mammals. No doubt these caches were specifically intended for storing little auks and their eggs and/or for preparing *kiviaq*. The dwellings also indicate a particular mode of settlement and social group configuration. Most are simple shelters in the form of a paved floor enclosed by low stonewalls with a small opening in one side (Fig. 2). With an average floor area of only 2.8 m^2^ (*n* = 17), they are clearly intended for a single or a couple of individuals and too small to house a regular family unit. The archaeological remains in Qoororsuaq thus emerge as a material reflection of the historical accounts of how (in the recent past), especially elderly people and children, too frail to join the hunt with their extended family, would separate from the group to settle at the little auk colonies in summer (Johansen 2013). Here, they were capable of providing for themselves throughout the little auk breeding season (early May–mid-August), and they were able to cache vast quantities of birds and eggs to be consumed by the whole settlement unit when re-united for the winter. The two winter houses, however, also testify to settlement outside the breeding season of the little auks. Close to these houses, two fox traps and a cairn row, presumably for snaring hares, were also recorded. Thus, it would seem that a small group of people at some point wintered in Qoororsuaq, possibly with the aim of trapping foxes. Due to the little auks, Qoororsuaq houses an impressive Arctic fox population (Lyngs 2011), and during the Thule Station Period (1910–1953), when fox fur represented great value, this may have made wintering at the site a feasible option. Located far from present day settlements, the little auks of Qoororsuaq are no longer directly exploited by humans. However, as a result of the lush vegetation caused by the fertilization from the little auks, Qoororsuaq currently houses a very large muskox population (see Fig. 6c). As revealed by the *Piniriarneq* project (see Flora et al. 2018), the valley has thus become an important muskox hunting site for Savissivik hunters. **Figure:** 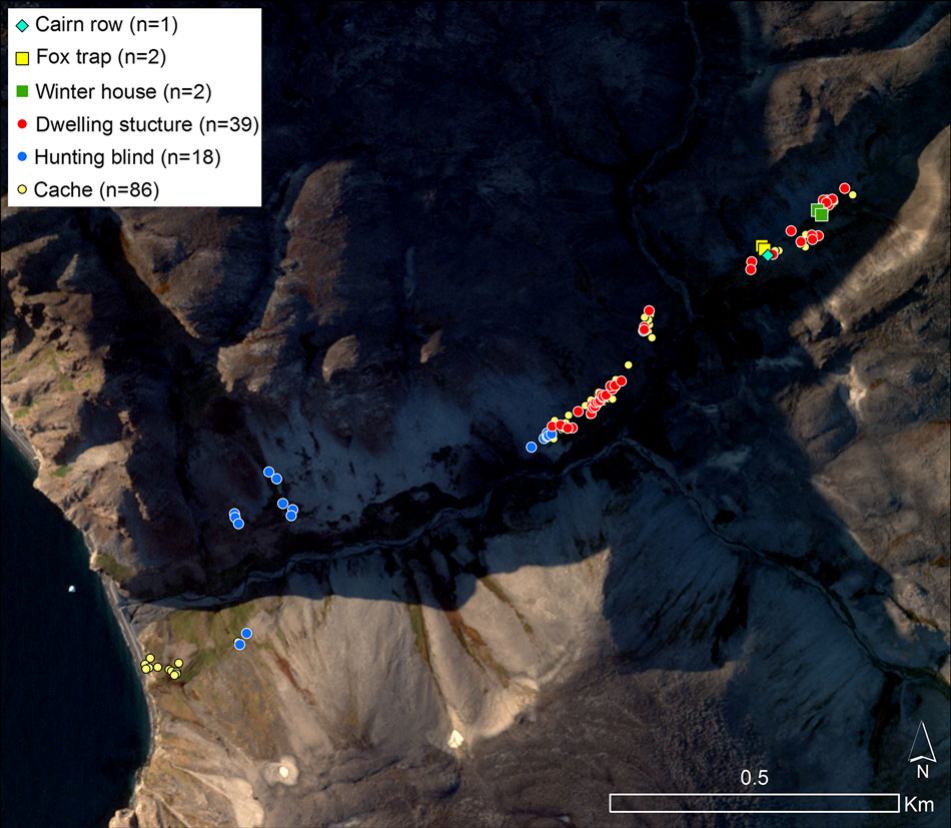 *Map of archaeological features recorded in the valley of Qoororsuaq on backdrop of a satellite image from 24.08.2015 (European Space Imaging GmbH/Space Imaging Middle East and DigitalGlobe, Inc.). The frequency of the different feature types is indicated in the legend. The grey-white slopes on both sides of the valley correspond to the breeding area of the little auks* **Figure:** 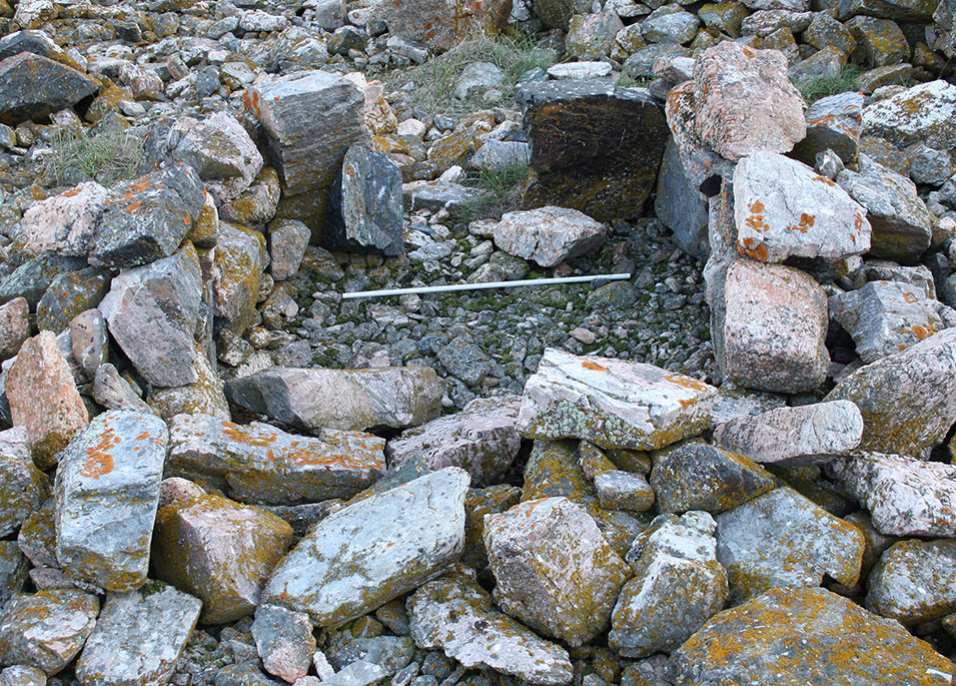 *Typical dwelling structure from Qoororsuaq—a small shelter consisting of a paved floor enclosed by low stonewalls with a small opening in one side. The white rod is 1 m long (Photo: Kasper Lambert Johansen)*	

#### Sustaining the Arctic fox population

The role of the little auk in the human history of the area is also a story of how it has indirectly benefited human societies. Consider the Arctic fox; in many parts of the Arctic, the northern-collared lemming (*Dicrostonyx groenlandicus*) constitutes the main prey of the Arctic fox, and the sizes of Arctic fox populations have been shown to follow the large natural fluctuations in lemming populations (Angerbjörn et al. 1999; Dalerum and Angerbjörn 2000). However, with the Humboldt Glacier defining the southern limit of lemmings in Northwest Greenland (Vibe 1990), the lemming is absent in Thule. Instead, the massive little auk colonies play a major role in sustaining the large Thule Arctic fox population. In a study of Arctic fox diet, Kapel (1999) found the stomach contents of foxes from Siorapaluk to be dominated by remains of little auks and their eggs, even though the examined foxes were trapped in winter. In summer, the little auk colonies abound with foxes, which are providing for their cubs and caching food for the winter (Lyngs 2011). “As is well known, foxes follow the little auks” (Rasmussen 1921, p. 541, author’s translation). In winter, there is a large in-flow of foxes to human settlements, making them readily available for harvesting. These likely represent a population surplus from summers spent at the little auk colonies.

From at least the 8th century AD and until the historical period, the Arctic fox has been an important source of clothing and food for both pre-Inuit and Inuit communities. Faunal assemblages from Late Dorset (8th to 13th century AD) and Ruin Island Phase (13th–15th century AD) sites always include Arctic fox, and at some sites in the area Arctic fox constitutes one-third to two-thirds of the total number of identified bone specimens (Appelt et al. 1998; Bendix 2000; Monchot and Gendron 2010; Monchot and Gendron 2011, Table 1; data compiled by Gotfredsen et al. 2018). Analyses of ethnographic collections also document an extensive use of fox fur in the Inughuit clothing of last century (Box 2). Especially during the Thule Station Period (1910–1953; Grønnow 2016), the Arctic fox became a very important resource in Avanersuaq. Trade in fox skins, which had a high international market value at the time, constituted the single most important factor in ensuring the financial viability of the station, and in funding the famed Thule Expeditions (e.g. Müntzberg and Simonsen 1996). For the Inughuit, who procured the fox skins, the trade with the station meant access to western commodities (e.g., guns, ammunition, traps, wood, tobacco, coffee, and sugar), and for decades, trapping of foxes was intensified throughout the region (see Hastrup et al. 2018; Flora et al. 2018). By providing an ecological foundation for the large Arctic fox population, the little auk was presumably a key player in this important fox fur trade.

**Table:** 

**Box 2 **Fur and feathers from the ethnographical collections	
Going through the ethnographical collections of clothing from across the Arctic, housed at the national museums of Greenland and Denmark, the Avaneruaq area is singularly unique, as it has a particularly high percentage the dress components made from fox or bird pelts (http://skinddragter.natmus.dk/). While the bird pelts were almost exclusively (eight out of nine pieces) used for inner parkas, the dress parts made from fox pelts were more diverse, i.e. used in men’s, women’s and children’s parkas, women’s short trousers, edge pieces, and loose hoods. Of the 25 outer parkas collected from Avanersuaq, there were nine of blue and white fox skin, nine of seal skin, three of dog skin, three of caribou, and one made of polar bear. The remarkably high number dress pieces made of fox pelts in the Avanersuaq probably reflects both the abundance of foxes in the area (likely resulting from the high density of little auks), and the time of acquisition of the ethnographical collections. Most pieces were acquired in the period of the Knud Rasmussen’s Thule Station (1910–1953), when fox-trapping was at its maximum. Within that 43-year period, harvest records were kept for 37 years. These describe an annual trade to the Station that averaged 1010 fox pelts, varied from 350 to 2100, and totalled 37 418 pelts (Hastrup et al. 2018). The constant high number of foxes trapped and traded over 37 years indicates that the Avanersuaq fox population was substantial and the carrying capacity high. **Figure:** 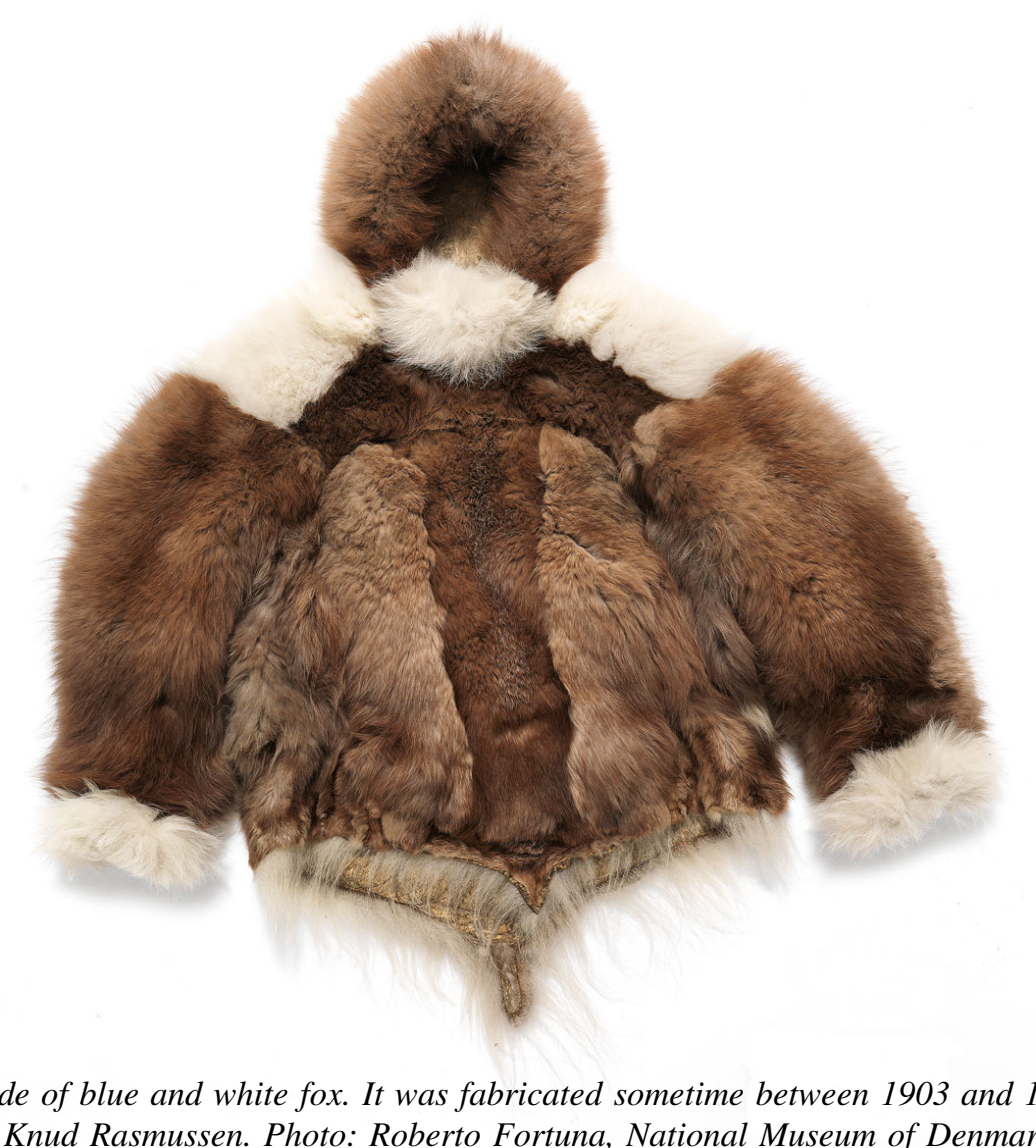 *Outer parka from Avanersuaq made of blue and white fox. It was fabricated sometime between 1903 and 1928, when it was donated to the National Museum of Denmark by Knud Rasmussen. Photo: Roberto Fortuna, National Museum of Denmark* **Figure:** 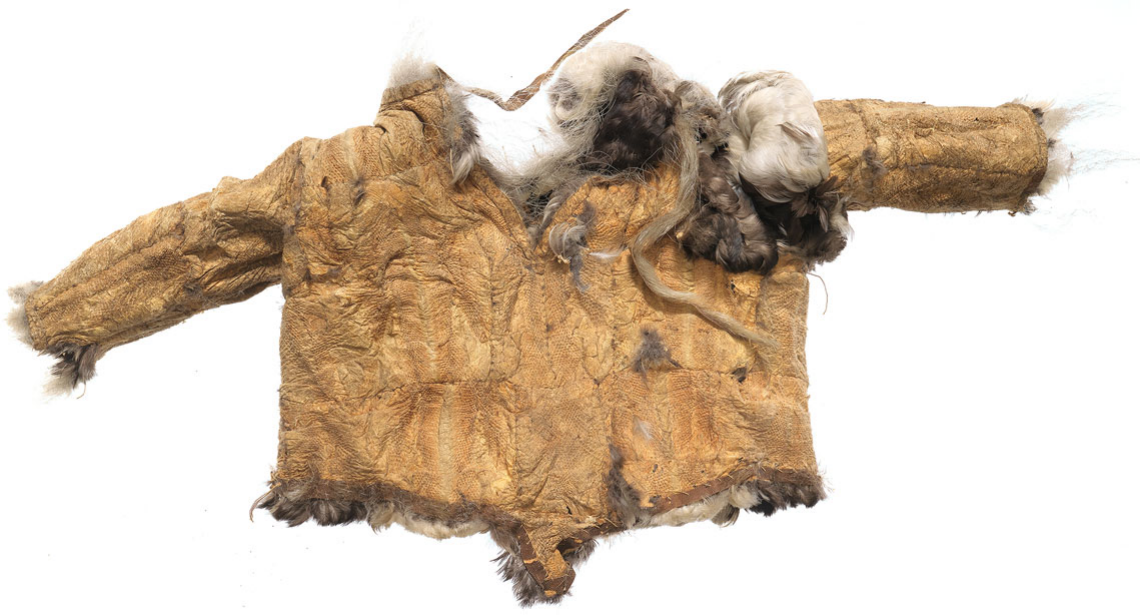 *Child’s inner parka from Avanersuaq made of little auk skin, worn with the feather side towards the body. It was made between 1910 and 1921, when it was donated to the National Museum of Denmark by Navarana or Peter Freuchen. Photo: Roberto Fortuna, National Museum of Denmark*	

## Discussion

We have illustrated how the little auk creates cohesion between land and sea and within human societies at both family and community level, and we have provided a comprehensive analysis of the substantial direct and indirect importance of the little auk in the NOW region. Although not a large source of meat today, the harvest remains much valued. At times in the past, the little auk was a key back-up resource enabling human survival in the area (Johansen 2013), and maybe at times simply a preferred resource. The large year-to-year variation observed in recent little auk harvest statistics may suggest that it still plays some role as a back-up resource. Similar to other seabird species, the reproductive capacity of the little auk is low, but its abundance has compensated and sustained their numbers despite an unregulated harvest, which is as large as needed. In contrast to the little auk, past harvesting techniques for other seabirds in the NOW region like Brünnich’s guillemot (*Uria lomvia*) was more time-consuming, which naturally restricted the numbers taken, until shotgun use became common and protective regulation was needed to maintain the populations (e.g., Kap York Stationen Thules Love af 7. juni 1929, Merkel et al. 2014). The fact that the little auk is harvested by ipoq, and not a noisy shotgun, is also vital to the sustainability of the harvest that takes place in the breeding colonies, which are vulnerable to disturbance. In the Faeroe Islands, the Atlantic puffin (*Fratercula arctica*), another alcid seabird, has for centuries been harvested in its breeding colonies with a “fleygastong”—a tool much resembling the ipoq. Key to sustaining the Faeroe Island harvest was a unique socially regulated system with ownership of bird colonies, which had the effect of preventing overharvesting (Nørrevang 1986). In Thule, there is no ownership to little auk catch sites and the harvest is often shared within and between communities in the region. The harvest has important social and cultural bearings as it can be practiced without costly equipment and has no gender and age discrimination in contrast to the dominant hunt for marine mammals. Thus, it can be argued that the little auk, in the past as well as in the present, acts as a social engineer, structuring social relations, and adding cohesion to the social fabric both on a local and regional scale.

On an ecosystem level, the little auk ensures that the riches of the North Water polynya are not confined to the marine environment, but also spills onto land. Also in that sense, the little auk weaves the whole region together. It is well known that seabirds can act as important resource linkers, transporting nutrients from sea to land via their guano (Sekercioglu 2006; Zwolicki et al. 2013), and on Arctic soils, generally poor in nutrients, there are several examples of such transport resulting in significant vegetation impacts (e.g., Croll et al. 2005). Gonzales-Bergonzoni et al. (2017) found little auk colonies in the NOW region to profoundly alter local freshwater and terrestrial ecosystems by providing nutrients, massively enhancing primary production, and changing chemical properties. Though increasing primary productivity, the high guano load in freshwater was also found to cause hypereutrophy and low pH, resulting in a reduced freshwater biodiversity. Based on elevated δ15 N in marine-derived nutrients, Gonzales-Bergonzoni et al. (2017) estimated that nutrients from little auk guano fueled more than 85% of terrestrial and aquatic biomass in little auk influenced areas. However, what really sets the little auks of NOW apart is the unprecedented scale at which the impact takes place. We estimate here that the little auks of NOW each year deposit approx. 3500 tons of N on land, and another study (Davidsson et al. 2018) indicates that the transport has been on-going for at least 4000 years, although the degree of variation in magnitude over time is unknown. Many seabird species, e.g., Brünnich’s guillemot (*Uria lomvia*), breed on cliffs facing the sea, which means that most of the guano falls on or seeps directly back into the sea (Stempniewicza et al. 2007). However, the little auk colonies of NOW are located up to 11 km inland from the coast and this means that large terrestrial areas are affected. We estimate that the little auk has transformed more than 200 km^2^ of otherwise barren ground into lush vegetated areas, creating landscape heterogeneity and changing the face of the whole region. Thus, in the NOW area, the little auk is an important ecosystem engineer, creating foraging habitats for a range of other species, including species important to human communities, both in the past and in the present.

All these ecosystem services of the little auk depend on their role as consumers of zooplankton in the marine environment. Karnovsky and Hunt (2002) estimated the total carbon flux to 60 million breeding little auks in the North Water to 147 400 mt C during the breeding season, and suggested that they locally and temporarily deplete the zooplankton population. This massive reliance on zooplankton makes the little auk vulnerable to changes in the marine ecosystem affecting zooplankton abundance, community composition, and accessibility (Marchese et al. 2017). Thus, current climate changes and associated warming of the sea have been raised as a concern for the little auk, as this might lead to a zooplankton community with predominance of more boreal species that are much smaller and less rich in lipids (Stempniewicza et al. 2007). In the Greenland Sea, little auks have been shown to spend more time foraging under warmer water conditions, presumably due to such differences in the zooplankton community (Welcker et al. 2009b). Other studies have, however, demonstrated that the little auk has some capability of adapting to such changes (Grémillet et al. 2012, 2015). In the NOW area, the largest potential threat to the little auk population in the future may be increased food competition from the recovering population of bowhead whales (*Balaena mysticetus*) (Rekdal et al. 2015) and from capelin (*Mallotus villosus*) that seem to be expanding northwards in response to climate change (Rose 2005; Ingvaldsen and Gjøsætter 2013).

## Conclusion

The ecosystem services of the little auk go far beyond the benefits of the direct harvest and include both a role as ecosystem engineer and as social engineer. In the NOW region, it is a species of significant ecological and cultural importance, and although there is currently no evidence of a population decline, the potential impact of climate change on little auk populations is certainly an area that warrants further study.

## Footnotes

1 http://uknea.unep-wcmc.org.

2 http://svalbardflora.no/index.php?id=296.
